# The Constitution of the Local Centre

**Published:** 1918-08-24

**Authors:** 


### THE MATRONS' AND SISTERS' DEPARTMENT.

### THE CONSTITUTION OF THE LOCAL CENTRE.

August and September bring an interval of quiescence to all societies, but that does not mean that the thinking part of the machinery is allowed to rest. On the contrary, during recess, while on moor and rocks the workers are taking in fresh energy, new plans often ripen, and minds set free from routine work turn eagerly in the direction of realising ideals in the days to come.

It is no secret that the College of Nursing has many important constructive matters on hand. The unparalleled rapidity with which it has taken root in the country at large is to be allowed no slackening, and already preparations are on foot in many directions for the formation of fresh Centres. The procedure which guides the College in its steady advance from headquarters to the outer ring wherein alone its full strength can be found and maintained is admirable in simplicity and elasticity. At first sight the business of forming a new Centre may appear to those who have had little experience in such matters quite overpowering. But when the successive steps on the road to affiliation are reviewed it will be seen that no superhuman powers are required for the business, only a little system, a little perseverance, a spirit of what may be called inclusiveness in opposition to the aloofness sometimes cultivated in institution life.

The Local General Committee is the backbone of the Centre while it is in process of consolidation. It is quite possible to form a Centre in places which can boast but a moderate section of College members. But the ability to get together a really representative committee of matrons, sisters, and nurses from the town and surrounding district is an essential preliminary to success. Representatives of all the major institutions, whether voluntary, military, municipal, or under the Local Government Board, ought to be secured, together with the heads of such responsibly managed nursing institutes, homes, and nursing charities as lie within the radius. The Matron or Head Sister of the asylum or mental hospital should also be included, for mental nurses, even when fully qualified medical and surgical nurses, tend to be too much isolated from the rest of the profession.

It is an important thing in forming committees of busy women to recognise the advantages of alternative representation. It is a principle already recognised by the Jubilee Institute and also by the College of Nursing so far as meetings of Local Representatives are concerned, and it might well be extended to purely local meetings of committees, attendance at which is often quite impracticable even for those most interested. If this principle were adopted, each matron or other official invited to serve on the General Committee would be asked to select a colleague to represent her should she be unable for any reason to attend herself. In this way a wider circle of women interested in the business would be secured, and the difficulty of making sure of a really good attendance would be overcome. A great deal of care should be expended on the formation of this General Committee, and it may be said at once that if the concurrence of the major nurse-training schools cannot be obtained it is a sure sign that the locality is not yet ripe for the formation of the Centre. The impulse to coalesce must come from those best qualified to lead in nursing affairs.

When this General Committee has been once formed on satisfactory lines the Centre may be launched. The Organising Secretary, Miss Cowlin, who gives her time almost exclusively to the business matters involved in the Centres, can be invited to come down and help the committee in their first step, the calling of a meeting for the formal inauguration of the Centre. The Hon. Arthur Stanley has done invaluable service for nurses by lending his support to such meetings, and has consented to address meetings in nearly every locality in which a Centre has been formed. The co-operation of the Mayor and other municipal personages ought also to be secured, for the formation of a Local Centre for nurses is a matter of public interest which cannot be regarded purely and simply from the standpoint of nurses. From the beginning the note struck must be significant of progress, of a wider outlook affecting the entire community rather than that of a trade union pledged to purely class interests.

